# Temperate silvopastures provide greater ecosystem services than conventional pasture systems

**DOI:** 10.1038/s41598-023-45960-0

**Published:** 2023-10-31

**Authors:** Helen C. S. Amorim, Amanda J. Ashworth, Peter L. O’Brien, Andrew L. Thomas, Benjamin R. K. Runkle, Dirk Philipp

**Affiliations:** 1 USDA ARS Poultry Production and Product Safety Research Unit, 1260 W. Maple Street, Fayetteville, AR 72701 USA; 2 Department of Crop, Soil, and Environmental Sciences, University of Arkansas, 115 Plant Sciences Building, Fayetteville, AR 72701 USA; 3 USDA ARS National Laboratory for Agriculture and The Environment, 1015 N. University Blvd., Ames, IA 50011 USA; 4 Division of Plant Science and Technology, Southwest Research Extension and Education Center, University of Missouri, Mt. Vernon, MO 65712 USA; 5 Department of Biological and Agricultural Engineering, University of Arkansas, 231 ENGR Hall, Fayetteville, AR 72701 USA; 6 Division of Agriculture, Department of Animal Science, University of Arkansas, AFLS B114, Fayetteville, AR 72701 USA

**Keywords:** Carbon cycle, Climate-change mitigation, Ecosystem services, Environmental chemistry, Environmental impact

## Abstract

Management and design affect systems’ ability to deliver ecosystem services and meet sustainable intensification needs for a growing population. Soil–plant–animal health evaluations at the systems level for conventional and silvopastoral environments are lacking and challenge adoption across temperate regions. Impacts of silvopasture on soil quality, microclimate, cattle heat stress, forage quality and yield, and cattle weight gain were compared to a conventional pasture in the mid-southern US. Here, we illustrate silvopastures have greater soil organic carbon, water content, and overall quality, with lower temperatures (soil and cattle) than conventional pastures. Forage production and cattle weight gains were similar across systems; yet, conventional pasture systems would need approximately four times more land area to yield equivalent net productivity (tree, nuts, forage, and animal weight) of one ha of silvopasture. Temperate silvopastures enhanced delivery of ecosystem services by improving soil quality and promoting animal welfare without productivity losses, thus allowing sustainable production under a changing climate.

## Introduction

Silvopasture is the integration of trees, forage, and livestock on the same piece of land, which diversifies production compared to conventional monocropping pasture systems and provides a range of ecosystem services^1^, including enhanced C sequestration, nutrient cycling, and water retention, improved animal welfare, conservation of biodiversity, and greater aesthetical value^1–5^. As such, silvopasture stands as a promising practice to meet global initiatives of promoting food security and the sustainable use of natural resources (https://sdgs.un.org/goals) and mitigation of greenhouse gas (GHG) emissions against the backdrop of 60% increase in sustainable food production by 2050^2^.

The capacity of silvopastures to deliver more and, in some cases, enhance ecosystem services, is typically associated with environmental benefits and greater system-wide stability in the face of climate change^3^. Carbon sequestration is a primary regulating service of silvopastures, owing to increased aboveground biomass and the presence of deep-rooted perennial grasses and trees^4,5^. Consequently, it is estimated that silvopasture systems can sequester 0.55–1.9 Mg ha^−1^ C per year, and the conversion of degraded and abandoned lands into silvopasture globally has the potential to assimilate 26.6 gigatons of CO_2_ equivalent by 2050^6^. Nevertheless, the management of silvopasture systems is complex, and employing proper tree and grazing intensity, the optimum combination of trees and forages, as well as proper nutrient management^3^, is critical to enhance C sequestration and nutrient cycling in silvospastures^7–10^.

System design and forage selection (e.g., cool- or warm-season grasses and/or leguminous species) can also impact the ability of silvopastures to provide food, feed, and fiber, as the presence of trees modifies the availability of light, water, and nutrients, creating competition for resources^11^. Forage production can be comparable between silvopasture and conventional pasture systems^12^, or higher in conventional systems^13,14^, while animal weight gains are oftentimes unaffected by the presence of trees. This lack of response in animal productivity is intriguing, since silvopastures can regulate the microclimate and create a cooling effect^15^, thus reducing animal heat stress and positively affect weight gains relative to conventional pasture systems^16,17^. Because system design and site-specific conditions also affect forage quality and grazing patterns^18–20^, livestock performance in silvopasture systems can be affected, but this deserves further investigation.

The integration of trees and pastures can enhance soil biodiversity^21^, increase soil aggregation and water infiltration, and reduce soil erosion and nutrient losses^22^. As such, silvopasture systems are expected to improve soil functioning (i.e., soil quality) compared to conventional pastures^23^, while promoting more efficient land use^24,25^. Using soil health assessment tools coupled with productivity metrics [e.g., land equivalent ratios (LER)]^26^ allows for a holistic evaluation of ecosystem services delivered by silvopastures and may promote their adoption across temperate regions. The Soil Management Assessment Framework (SMAF)^27^ evaluates dynamic soil properties as affected by management practices and has been used to evaluate soil quality in tree-based systems^23,28^. However, evaluations of ecosystems services at the system-level, comparing silvopasture and conventional pasture systems, have not been systematically conducted in temperate, subtropical humid systems to date.

Thus, this study aims to assess delivery of supporting, regulating, and provisioning services of mid-southern US silvopasture and conventional pasture systems by means of evaluating differences in soil properties, microclimate, forage production and quality, cattle temperature, cattle weight gains, and SMAF soil quality indices. We hypothesized that silvopasture (compared to conventional pasture) will improve soil quality (H1); increase forage yields and lower cattle temperatures, which will contribute to increased cattle weight gains (H2); and that forage quality and yields of cool- and warm-season species will differ between systems (H3).

## Results

### Trends in soil quality across temperate systems

**Figure 1:**
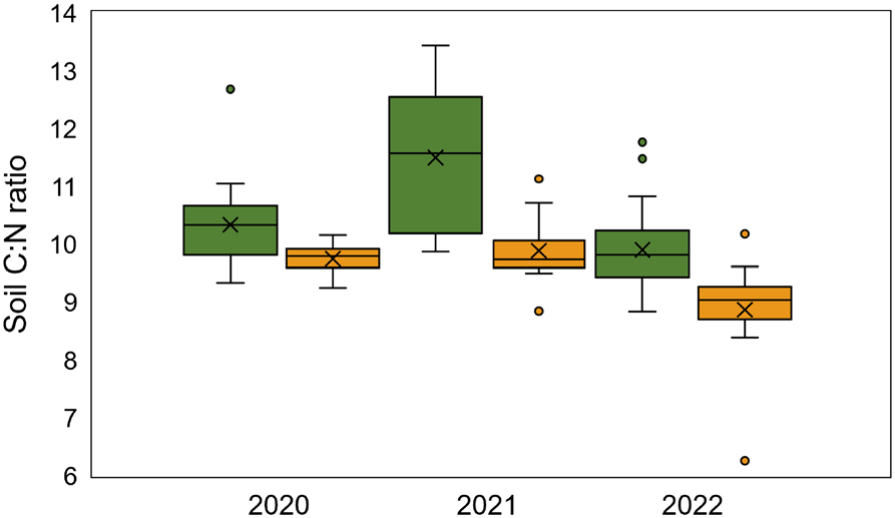
Soil C:N ratios in silvopasture and conventional pasture systems. Boxplot showing interactive effects of system (silvopasture and pasture) and year (2020, 2021, and 2022) on soil C:N ratios at the 0–15 cm soil depth in silvopasture and conventional pasture systems in Fayetteville, AR (*n* = 15). The line and the × inside the boxplot indicate the median and mean values, respectively. The top and bottom of boxplot correspond to the 25th and 75th percentile, respectively, and whiskers extend to 1.5 times the interquartile range.

Soil C:N ratios changed between systems and over time (Supplementary Table 1), with greater C:N and SOC (16.1 vs. 13.7 g kg^−1^) in the more complex silvopasture system. The highest mean C:N ratio occurred in the silvopasture in 2021 (11.46), reflective of high SOC (16.29 g kg^−1^) coupled with lowest mean N level (1.42 g kg^−1^) in that year (Fig. 1). In turn, the lowest mean C:N ratio occurred in the conventional pasture system in 2022 (8.86), reflecting the lowest mean SOC content (13.35 g kg^−1^) and high soil N level (1.52 g kg^−1^). Bulk density, SOC, soil pH, Ca, and K contents differed between systems, whereas soil pH, P, N, Mg, and S contents varied by year (*p* < 0.05; Supplementary Table 1). Electrical conductivity (EC) did not differ between systems or by year (*p* > 0.05).

At the 0–15 cm soil depth, mean SOC content in the silvopasture was 16.1 g kg^−1^, 18% higher (*p* < 0.05) than in the conventional pasture system (13.7 g kg^−1^; Table 1)^24,33,34^. Mean bulk density at the 0–15 cm depth in the silvopasture (1.22 g cm^−3^) was 6% lower than the conventional pasture (*p* = 0.008; 1.30 g cm^−3^). Mean soil C and N stocks did not differ between systems (*p* = 0.22).

**Table 1:** SOC, soil N, and soil chemical properties via Mehlich-3 measured at the 0–15 cm soil depth in silvopasture and conventional pasture systems in Fayetteville, AR, per system (silvopasture and pasture) and year (2020, 2021, and 2022) (mean ± standard error; *n* = 15), averaged across years and systems, respectively.

Effect	Soil properties^†^									
	SOC	N	C:N	pH	EC	Ca	Mg	K	P	S
	g kg^−1^				µS cm^−1^	mg kg^−1^				
System										
Silvopasture	16.1 ± 0.7 a^‡^	1.5 ± 0.06 a	10.6 ± 0.1 a	6.7 ± 0.05 a	51.2 ± 2.5 a	1451.9 ± 65.6 a	52.9 ± 3.2 a	75.2 ± 5.8 b	44.3 ± 3.6 a	15.1 ± 0.5 a
Pasture	13.7 ± 0.8 b	1.4 ± 0.06 a	9.5 ± 0.2 b	6.4 ± 0.05 b	43.8 ± 2.6 a	986.7 ± 72.4 b	56.7 ± 3.5 a	96.1 ± 6.4 a	43.6 ± 4.1 a	15.4 ± 0.5 a
Year										
2020	15.1 ± 0.7 a	1.5 ± 0.06 ab	10.0 ± 0.1 b	6.7 ± 0.06 a	46.8 ± 2.7 a	1342.9 ± 82.1 a	61.9 ± 3.7 a	86.7 ± 7.5 a	50.3 ± 3.9 a	17.9 ± 0.7 a
2021	14.9 ± 0.7 a	1.4 ± 0.06 b	10.7 ± 0.1 a	6.5 ± 0.06 a	50.7 ± 2.8 a	1087.3 ± 83.5 a	46.4 ± 3.8 b	76.8 ± 7.6 a	44.5 ± 3.8 ab	15.1 ± 0.7 b
2022	14.6 ± 0.7 a	1.6 ± 0.06 a	9.4 ± 0.1 c	6.3 ± 0.06 b	45.1 ± 2.8 a	1227.8 ± 83.4 a	56.2 ± 3.7 ab	93.4 ± 7.6 a	37.0 ± 4.0 b	12.7 ± 0.7 c

^†^*SOC* soil organic C; *C:N* carbon-to-nitrogen ratio; *EC* electrical conductivity;

^‡^Means followed by the same letter do not differ (*p* > 0.05).

Additionally, the silvopasture system had higher soil Ca content and pH (*p* < 0.05; Table 1), which is evidence of the legacy liming effect from poultry litter applications^29^, whereas soil K content was 28% higher in conventional pasture systems (*p* < 0.05). A trend of decreasing soil pH, and P and S contents was observed from 2020, and the lowest Mg content occurred in 2021.

Silvopasture had higher SOC, BD, and pH individual SMAF scores than the conventional pasture system, leading to a higher overall SQ Index of 78.8% (Table 2; *p* < 0.05), vs. 72.9% in the conventional pasture, and thus supporting the first hypothesis. The higher SOC score in the silvopasture reflects the increased SOC content in the 0–15 cm soil depth; in turn, the higher BD score was due to the lower BD under tree-pasture systems.

**Table 2:** Soil Management Assessment Framework (SMAF) individual scores and overall SQ index based on soil samples collected at the 0–15 cm depth in silvopasture and conventional pasture systems in Fayetteville, AR, in 2022 (*n* = 15).

System	SMAF scores^†^							
	SOC	BD	pH	EC	P	K	SQI	SQI (%)
Silvopasture	0.86 a^‡^	0.94 a	0.98 a	0.17 a	0.99 a	0.70 a	4.57 a	78.8 a
Pasture	0.75 b	0.82 b	0.93 b	0.13 a	0.99 a	0.73 a	4.22 b	72.9 b
*p-value*	0.045	< 0.0001	0.009	0.086	0.971	0.613	0.001	0.009

^†^*SOC* soil organic C; *BD* bulk density; *EC* electrical conductivity; *SQI* soil quality index;

^‡^Means followed by the same letter do not differ (*p* > 0.05).

### Cooling effect of silvopastures on microclimate and cattle temperature

**Figure 2:**
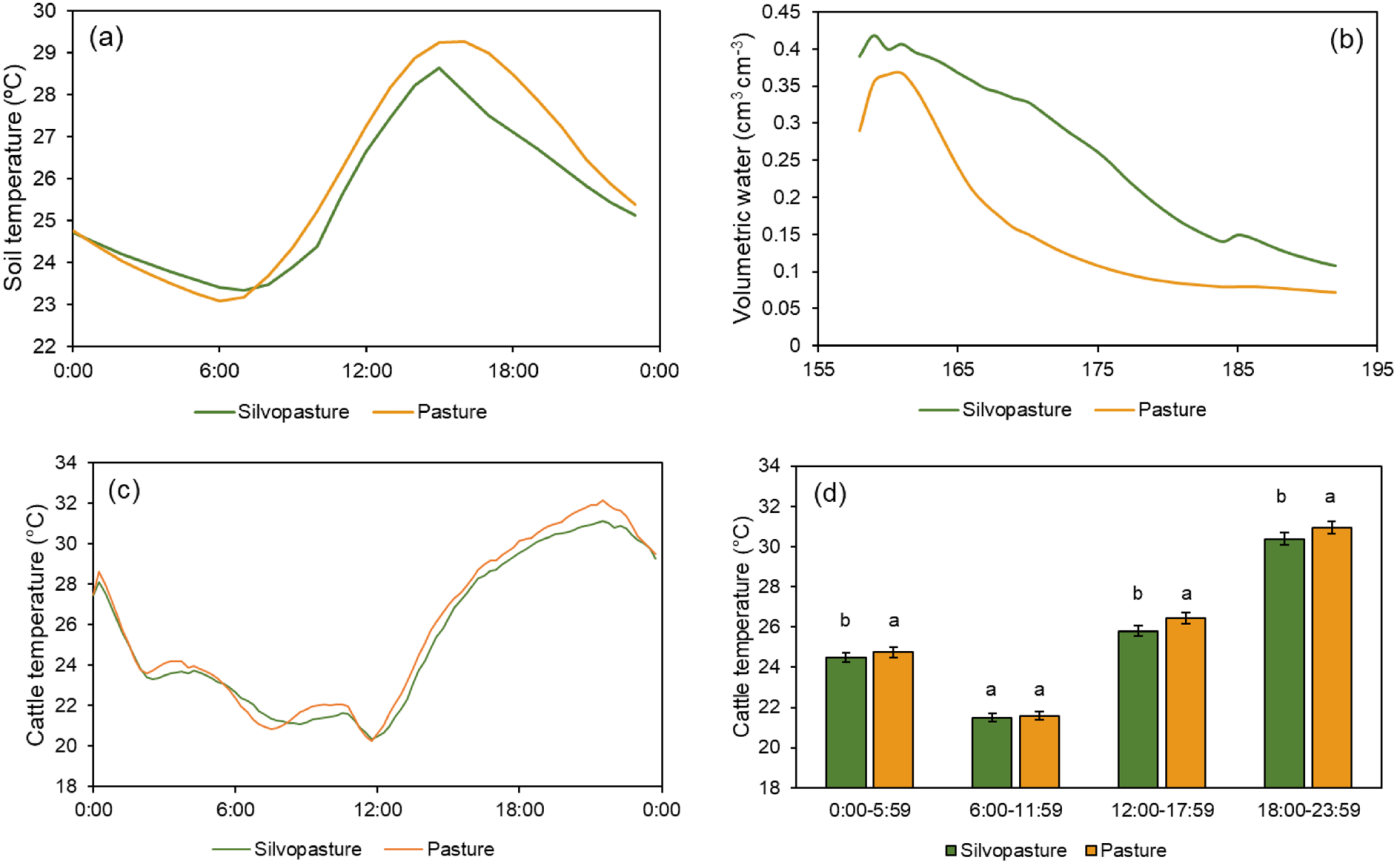
Microclimate and cattle temperature in silvopasture and conventional pasture systems. Mean soil temperature (**a**) and mean content of soil volumetric water (cm^3^ cm^−3^; **b**) at the 0–15 cm depth, and mean cattle temperature (°C) measured via sensors on cattle collars (*n* = 10; Model 3300LR, Lotek Wireless Inc., Newmarket, ON; **c**) and cattle temperature comparison per time interval (0:00–5:59, 6:00–11:59, 12:00–17:59, and 18:00–23:59; **d**) in the silvopasture and conventional pasture systems in Fayetteville, AR, measured between June 7, 2022 and July 11, 2022. Means followed by the same letter do not differ (*p* > 0.05) within time interval.

During the grazing window (June 7–July 11, 2022), mean soil surface temperature in the silvopasture (25.6 °C) was lower than the conventional system (25.9 °C; Fig. 2a; *p* < 0.05). Mean soil volumetric water content was 0.24 cm^3^ cm^−3^ in the silvopasture, or 51% higher than in the conventional pasture (0.16 cm^3^ cm^−3^; Fig. 2b; *p* < 0.05).

Mean collar cattle temperature in the silvopasture system was 25.5 °C, or 1.6% (0.39 °C) lower (*p* < 0.05) than the conventional pasture (25.9 °C). Cattle temperatures ranged between 20.3 and 31.1 °C in the silvopasture and between 20.2 and 32.2 °C in the conventional pasture system (Fig. 2c). The lowest daily temperatures were between 6:00 and 11:59, and the highest temperatures occurred between 18:00 and 23:59 (Fig. 2d). Mean daily temperatures were lower in the silvopasture than in the conventional pasture, except for the 6:00–11:59 interval, when mean temperatures did not differ *(p* > 0.05; Fig. 2d). Specifically, mean cattle temperatures in the silvopasture were 0.25, 0.64, and 0.57 °C lower than in the conventional pasture in the 0:00–5:59, 12:00–17:59, and 18:00–23:59 intervals, respectively (*p* < 0.05; Fig. 2d).

### Systems productivity and land use efficiency

**Figure 3:**
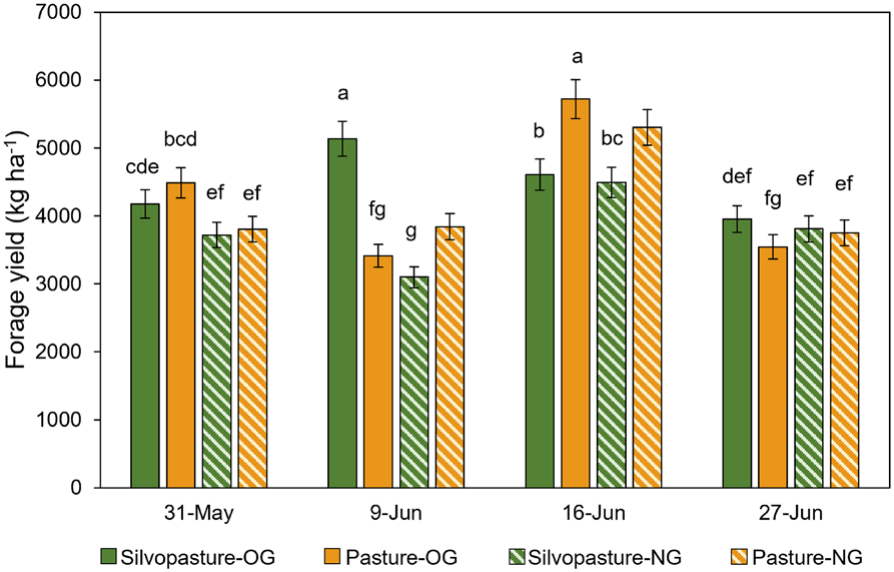
Forage mass in silvopasture and conventional pasture systems. Forage mass (kg ha^−1^) measured in the silvopasture and conventional pasture systems in Fayetteville, AR, per forage species [cool-season orchardgrass (OG) and native warm-season mix (NG)] and sampling dates (May 31 to June 27, 2022). Means followed by the same letter do not differ (*p* > 0.05).

At the systems-level, forage mass in the silvopasture (4,125 kg ha^−1^) and conventional pasture (4,234 kg ha^−1^) were not different (*p* > 0.05). Nevertheless, systems, forage species, and sampling dates interacted (*p* < 0.05). Mean forage mass values ranged from 3100 to 5137 kg ha^−1^ in the silvopasture and between 3413 and 5720 kg ha^−1^ in the conventional pasture, with a peak production on June 9 for the cool-season orchardgrass (*Dactylis glomerata* L., var. Tekapo)], and June 16 for the warm-season native mix (Fig. 3). On May 31, orchardgrass in the conventional pasture had 18–20% higher forage mass than the native warm-season grass mix in both systems, not differing from orchardgrass in the silvopasture. On June 9, orchardgrass in the silvopasture had 66% higher yield than the native mix in the same system, and 51 and 34% higher yields than orchardgrass and the native warm-season grasses in the conventional pasture, respectively. On June 16, however, both orchardgrass and the native mix in the pasture had the highest forage mass of all sampling dates, not differing from the forage mass of orchardgrass in the silvopasture on June 9. In the last sampling date (June 27), forage mass was similar across systems and forage species, varying between 3545 and 3955 kg ha^−1^.

Similarly, most forage composition variables were affected by the interaction of systems, forage species, and sampling dates (*p* < 0.05; Supplementary Table 2), except for C and ash contents. Overall, forage species grown in the silvopasture had the highest N, neutral- (NDF), and acid-detergent fiber (ADF), and crude protein contents, whereas C:N ratios, C, and lignin were the highest in the conventional pasture system across sampling dates. Specifically, warm-season native grasses in the silvopasture had the highest crude protein on May 31 and June 27 (12.3 and 9.5%, respectively), whereas on June 9 and 16, orchardgrass crude protein in the silvopasture (9.7 and 9.3%, respectively) was higher than in the native grasses in the same system (8.4 and 8.1%, respectively). Warm-season native grasses in the silvopasture had the highest ADF and NDF contents on June 16 (43.8 and 68.1%, respectively), although these values did not differ from the ADF and NDF contents observed on June 27 for orchardgrass grown in the silvopasture and the warm-season native grasses in the conventional pasture (Supplemental Table 2).

We did not observe a systems-level effect for mean cattle weight (*p* = 0.4563). In the silvopasture, mean weight of heifers was 497 kg after 36 days, corresponding to a mean 30 kg weight gain per animal, or 0.83 kg day^−1^. In the conventional pasture, after 36 days, mean cattle weight was 490 kg, corresponding to a 34 kg weight gain per animal, or 0.94 kg day^−1^. However, owing to the greater total area (4.25 ha), the cattle weight gain per area in the silvopasture (*p* = 0.0477; 7 kg ha^−1^) was lower compared to the conventional pasture (10.3 kg ha^−1^).

The LER of the silvopasture relative to the conventional pasture was 4.39 using forage mass (kg ha^−1^) as a metric of pasture productivity (Table 3), indicating that 3.39 ha more land would be needed to produce separately (e.g., tree stands and pasture in different areas) the same amounts of timber, nuts, and forage mass produced in one ha of silvopasture. Considering cattle weight gains (kg ha^−1^) as a metric of pasture productivity, the LER was 4.10 (Table 3). Similarly, such a number indicates that silvopasture and pasture only systems would need 3.10 ha more area to equal the tree yields and cattle weight gains of one ha of silvopasture.

**Table 3:** Silvopasture and conventional pasture yields, and individual components of land equivalent ratios (LER).

System component†	Silvopasture	Pasture	Forest reference	LER component
Pecan nuts (kg ha^−1^)	606		842^30^	0.72
Cottonwood DBH (cm)	26.1		35.6^31^	0.73
Sycamore DBH (cm)	15.7		23.0^32^	0.68
Northern red oak DBH (cm)	34.2		48.8^24^	0.70
Pine DBH (cm)	11.4		19.4^33^	0.59
Forage dry mass (kg ha^−1^)	4125	4234		0.97
			LER	4.39
Pecan nuts (kg ha^−1^)	606		842	0.72
Cottonwood DBH (cm)	26.1		35.6	0.73
Sycamore DBH (cm)	15.7		23.0	0.68
Northern red oak DBH (cm)	34.2		48.8	0.70
Pine DBH (cm)	11.4		19.4	0.59
Cattle weight gain (kg ha^−1^)	7.0	10.3		0.68
			LER	4.10

^†^*DBH* diameter at breast height (137 cm); cattle weight gain between June 6 and July 12, 2022.

## Discussion

Tree leaf litter and root sloughing associated with the long-term poultry litter applications contributed to the soil organic matter (SOM) build-up in the silvopasture over the 22 years of management^34–36^, now reflected in the greater SOC content relative to the conventional pasture system. Although the systems-level comparison occurred over a 3-year window, it should be noted that the conventional pasture has been a pasture for at least 20 years, but only intensively managed in the past 3 years to mirror the silvopasture management. While the results presented here may seem limited in terms of replication, the soil and climate conditions of this study site are representative of the mid-southern US (Ultisols in a humid subtropical climate—Cfa) and can scale across this region.

The silvopasture and conventional pasture systems present mean SOM contents of 3.22 and 2.74%, respectively^37^. While a 0.48 percentage-point difference may seem minor, this higher SOM content can play a critical role in soil water retention and overall system resiliency^38^. It is estimated that a 1% increase in SOM content can increase available water-holding capacity by 1.5–1.7%^39^. As such, the silvopasture and conventional pasture system can hold 42.3 and 36.7 m^3^ water per acre furrow slice, respectively. Thus, the 0.48 percentage-point higher SOM content in the silvopasture corresponds to a 15% greater water storage, or 14.0 m^3^ ha^−1^, allowing for greater resilience to drought and climate change. Long-term monitoring is needed, particularly for deeper soil layers, to assess SOC changes in the silvopasture system and its continued ability to support nutrient cycling and water retention.

Despite the greater SOC contents, SOC stocks did not differ between systems, a trend that has been recently observed by Veldkamp et al. (2023)^3^ when comparing grasslands and croplands to agroforestry systems in Germany. Upson et al. (2016)^40^ argue that SOC stocks in silvopasture topsoil can be affected more by forage than tree inputs; thus, increases in SOC stocks in silvopasture relative to pasture can be lower or not significant compared to other agroforestry systems (e.g., alley cropping)^5^. The SOC stock of 29.1 Mg ha^−1^ was slightly lower than global mean value of 35 Mg ha^−1^ presented by Shi et al. (2018)^5^, and lower than the reported mean SOC stock for temperate agroforestry systems (47 Mg ha^−1^) at the 0–20 cm layer^41^. Still, the value presented in this study is higher than that measured in 2016 (25.8 Mg ha^−1^) in the same silvopasture system^9^ indicating that SOC stock continues to increase in the topsoil. Furthermore, increased C and N sequestration rates demonstrate the potential of silvopasture systems to store C and N in above and belowground biomass^8^ in temperate, subtropical humid regions.

The trend of decreasing soil C:N ratios between 2020 and 2022 can be concerning, as it may reflect a decoupling in the C and N cycles in the silvopasture, i.e., disproportional increases in soil N compared to SOC, thus affecting SOM turnover and stabilization^42^. Indeed, lower mean soil C:N ratio in converted pastures (16.5) compared to converted silvopastures (18.4) were linked to increases in soil N, whereas SOC remained unaltered in the 0–30 cm layer^43^. Enhanced nutrient use efficiency through proper fertilization management is key to enhance nutrient cycling and abatement of GHGs in agroforestry systems^3^. Here, the observed reduction in soil C:N ratio can be attributed to C losses through microbial respiration in more warmer and humid conditions compared to other temperate systems, with selective N accumulation after continuous leaf and poultry litter inputs in the silvopasture^10^. As such, an imbalance between SOC and N contents can affect the ability of this system to retain C and other nutrients in soil, thus underscoring the need for long-term system evaluations.

The increased SOC content and lower BD in the silvopasture at the 0–15 cm depth led to higher SOC and BD SMAF scores and, consequently, greater soil quality than the conventional pasture system (Table 2). The milder microclimate created by the trees and the greater soil moisture may have altered microbial community and activity, thus slowing SOM decomposition compared to the pasture only system^21^. Increased SOM levels are generally linked with enhanced nutrient availability and soil fertility, improved soil structure, and greater water retention, microbial activity and diversity, ultimately leading to greater system productivity^44^. As such, the SMAF algorithm for SOC uses the “more-is-better” approach, reflective of the positive relationship between increased SOM levels and the ability of soils to function and support primary production^45^. Therefore, tree-based systems had greater SOC scores and soil quality than single use systems.

Other studies support our results on greater soil quality in silvopasture systems and demonstrate the positive impacts of agroforestry practices on SOC, nutrient availability, and soil biodiversity^46^. Poudel et al. (2022)^21^ showed that a 25-year old temperate hardwood silvopasture in the Southern Appalachian Ridge and Valley region, VA, had higher SOM, microbial biomass C, and enzyme activity than pasture without trees. Moreover, the authors showed that increases varied according to tree species, stand age, and system management. Subtropical agroforestry systems receiving cattle manure and phosphate rock applications had greater SMAF soil quality indices than sole forest and pasture, due to the enhanced SOC and microbial biomass C, higher soil fertility, and overall greater soil structure^23^, which highlights the importance of proper management of fertility and grazing intensity to improve, or even restore, soil health^47^.

Near surface soil temperatures in the silvopasture system were typically higher overnight and into the early morning, while they were lower during the warmest part of the day (Fig. 2a). This reduction in soil temperatures in late afternoon is typical of silvopasture sites^43,48^ as tree canopies intercept incoming radiation and reduce radiation available to heat the soil. Conversely, the warmer nighttime soil temperatures are likely due to the warming effect of the tree canopy trapping air and longwave radiation in the canopy. Soil water content remained higher in the silvopasture than the conventional pasture throughout the grazing period (Fig. 2b). This finding contrasts with some other work reporting lower soil water content under silvopasture that was likely due to increased water uptake by trees^49,50^. However, tree canopies shaded grasses and soil surface from incoming radiation, which appears to be a greater factor in regulating near surface soil water content. Notably, these measurements were made under the oak trees, which had the highest canopy density, and these effects may have been lessened in the species with less mature trees and lower canopy density.

In a similar pattern, mean cattle collar temperatures in the silvopasture were lower throughout the day than in the conventional pasture system (Fig. 2d). This cooling effect is critical under the current global warming scenario, as it reduces evapotranspiration in the soil–plant system, as noted above per the higher soil water content, while promoting animal welfare. The lower temperatures indicate that the microclimate created by trees can reduce cattle heat stress during warmer parts of summer days relative to pastures without trees. Indeed, cattle present a more even distribution and grazing patterns in silvopasture compared to conventional pastures^51^, although the relationship between reductions in heat stress and cattle performance is not well understood yet^16^. Providing appropriate levels of shade can help grazing animals to reduce the energy spent with thermoregulation, which may improve feed conversion and weight gains^17^.

Despite the improved soil quality and milder microclimate conditions in the silvopasture, mean cattle weight gains (kg day^−1^) and forage DM yields were similar between systems. Thus, we reject our second hypothesis. This result partially agrees with those from Kallenbach et al. (2006)^13^, who found that the presence of trees reduced forage yields by 20%, but increased crude protein and reduced acid- and neutral detergent fiber contents. Such improved forage quality in the silvopasture likely offset the lower forage yields, leading to equal daily weight gains for both silvopasture and conventional pasture systems (0.75 kg day^−1^). In the present study, it is possible that a similar process occurred: the overall higher nutritive value in both cool- and warm-season forages in the silvopasture contributed to cattle weight maintenance during the grazing period. Still, we acknowledge that a longer evaluation period may be needed to monitor variations in temperature and forage quality and capture effects on cattle weight gains in silvopastures across temperate, subtropical humid regions.

**Figure 4:**
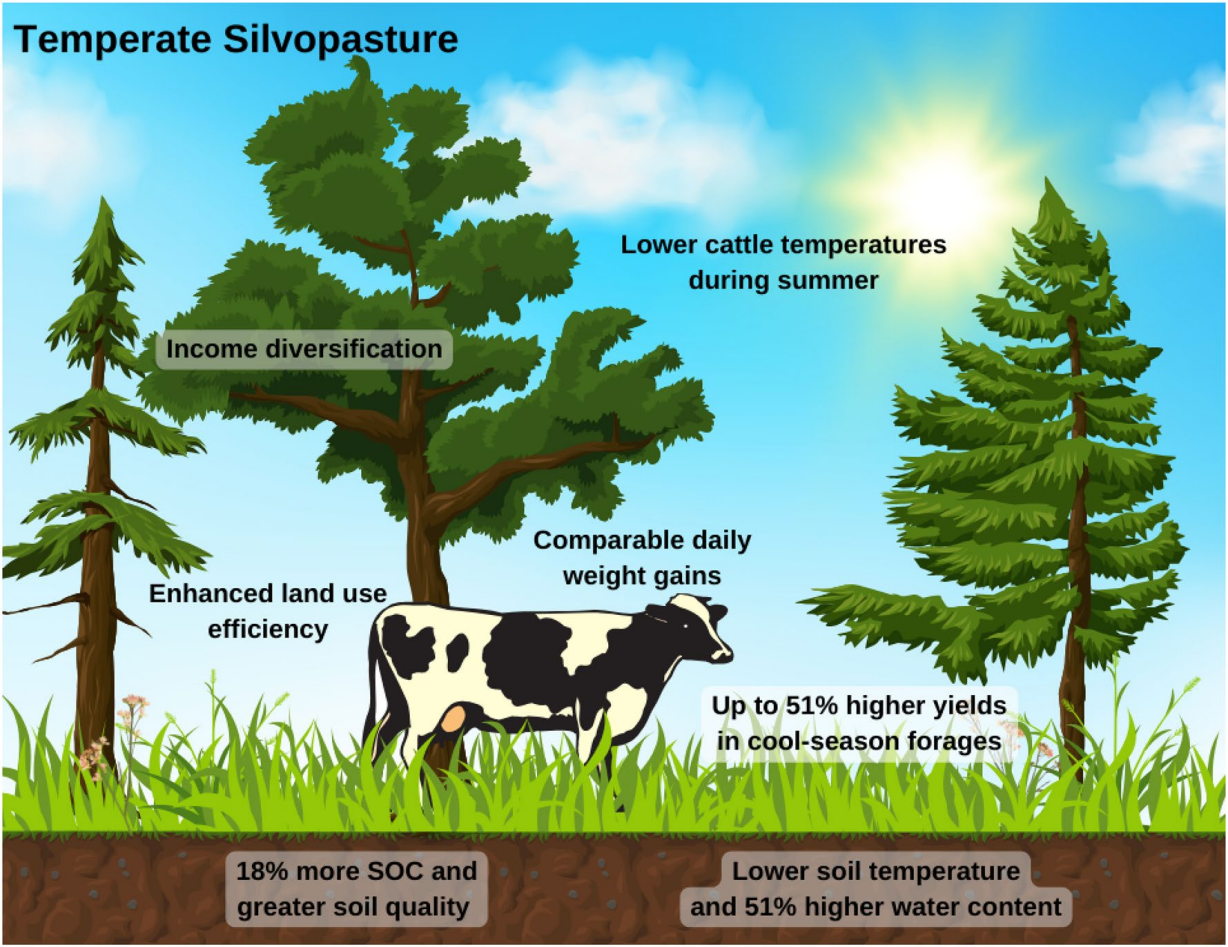
Ecosystem services in a temperate silvopasture system. Summary of supporting, regulating, and provisioning services provided by temperate silvopastures relative to conventional summer mob grazed systems.

The greater orchardgrass DM yields in the silvopasture and the native grass mix in the conventional pasture system (Fig. 4) illustrates C3 and C4 photosynthesis response differences to shading and microclimates. Orchardgrass (C3 photosynthetic pathway) often reaches light saturation at about 50% of full sun, whereas the native warm-season mix (C4 photosynthetic pathway) has light saturation at about 85%^52^. Therefore, shade provided by the trees can reduce production of C4 forages, whereas cool season C3 species can still thrive under 50% shade^53^. In contrast, warm-season forages are more drought tolerant and more productive during warmer months, which supports the overall higher yields of the native grass mix in the conventional pasture compared to the silvopasture system. Additionally, both warm- and cool-season forages possibly benefited from the higher soil K levels (Table 1) in conventional pasture, which may have contributed to improved water balance and plant growth under drier and warmer conditions.

Land equivalent ratios suggested that, despite the similar forage and cattle yields between systems (Table 3), silvopasture over-yielded the conventional pasture system. The critical value of LER is 1; thus, values greater than 1 indicate that more land area of forest only or pasture only is needed to achieve the timber and forage, or timber and cattle yields (weight gain or meat) obtained in one hectare of silvopasture^26,54^. The values obtained in this study (LER > 4) are much higher than those compiled by Pent et al. (2020)^24^ (1.1–2.1). However, in that meta-analysis, the author only included agroforestry systems with one tree and one forage or cattle component, while in the present study, we added all the tree products present in this system to the forage or cattle component. If separated by tree stands, we would obtain LER values of 1.56–1.7 and 1.27–1.41 when adding tree + forage and tree + cattle component, respectively (Table 3), thus aligning with those values in the literature. Using cattle weight gains as a metric of productivity usually results in lower LER values than forage yields^24^. This result is largely because (1) reductions in forage yields in silvopasture compared to pastures are minimal, especially for cool-season forages and (2) cattle weight gains in conventional pasture systems can be higher than in silvopastures. Indeed, mean forage dry mass in the silvopasture was only 3% lower than the conventional pasture (LER component of 0.97; Table 3), whereas mean cattle weight gain per area was 32% lower than the conventional pasture (LER component of 0.68).

A recent review from Smith et al. (2022)^55^ demonstrated the perceived benefits of silvopasture by producers in the US and shows that income diversification and promotion of animal welfare were the primary reasons fostering the adoption of silvopasture. Producers reported using silvopasture mostly as a complementary practice to their pasture management and indicated that lack of information is the one the primary challenges of silvopasture. Besides that, they argue that the perceived environmental benefits of silvopasture (e.g., C sequestration, pollution control runoff, and improvement of wildlife habitat) are not enough to compensate the additional costs and complexity related to system management. Indeed, the management of trees, forage, and cattle to optimize the use of resources while maximizing profit is challenging and requires technical guidance^17^. Long-term studies that evidence the environmental and economic benefits of silvopasture relative to conventional systems are critical to support the decision-making process regarding system management, and to foster the development of policies and further payments to farmers towards the adoption of silvopasture as a regenerative and profitable practice.

Payments for ecosystem services are expected to increase as governments commit to combating climate change and achieving net-zero GHG emissions by 2050^56^ and compliance markets expand. Here, the 3.3 Mg C ha^−1^ increase in SOC stock in the silvopasture between 2016 and 2022 would generate about US$ 8.7 ha^−1^ year^−1^ in C credits in the voluntary C market (US$ 4.3/ton CO_2_ equivalent for nature-based projects), or US$80–160 ha^−1^ year^−1^ in the compliance market (US$ 40–80/ton CO_2_ equivalent) as additional revenue. Moreover, improving soil health through conservation management can increase net farm income by US$ 162.5 ha^−1^^57^, evidencing economic gains of switching from conventional to conservation agriculture. As such, silvopasture is a climate-smart agricultural practice that goes beyond land conservation, promoting income diversification and increasing farm profitability.

Through an integrated approach, we quantified the benefits of silvopastures on soil quality, forage production, and livestock performance (Fig. 4), and that information can support the adoption of silvopasture systems in humid subtropical regions, or even assist their management towards greater productivity and profitability. We found that silvopasture creates multifunctional landscapes that enhance the delivery of a range of ecosystem services compared to conventional pastures. The supporting and regulating services, represented by greater SOC content, higher soil water content, and improved soil quality in the silvopasture underscore the environmental benefits of more complex and diverse systems over monocultures. The potential for C sequestration in these systems in both soil and tree biomass suggests it may be a valuable climate change mitigation practice. Similarly, the moderation of temperatures in both soil and cattle compared to conventional systems demonstrates the value of silvopastures as a climate change adaptation practice. Further, provisioning services, here measured as forage production and cattle weight gains, were similar between systems, suggesting that weight gains are also affected by forage quality, and that the integration of trees and pasture should cause no loss in global system productivity. The improved LER may be especially important in increasing adoption of this practice, since it justifies the more complex management required in silvopastures. Therefore, silvopasture systems allow for sustainable intensification in temperate, humid subtropical regions, and stands as a promising practice to mitigate the negative impacts of climate change while meeting growing global food, feed, and fiber production demands.

## Methods

### Site description

This study was carried out at the University of Arkansas Agricultural Research and Extension Center in Fayetteville, AR (36°50′ N; 94°100′ W, 382 m a.s.l., and 3.4% mean slope). The experimental area consisted of a 4.25 ha silvopasture and a 3.3 ha conventional pasture system. Soils in the silvopasture area are mapped as Captina silt loam (fine-silty, siliceous, active, mesic Typic Fragiudults), with some portions of Pickwick silt loam (fine-silty mixed, semiactive, thermic Typic Paleudults) and small areas of Nixa cherty silt loam (loamy-skeletal, siliceous, active, mesic Glossic Fragiudults) and Johnsburg silt loam (fine-silty, mixed, active, mesic Aquic Fragiudults)^58,59^. In the conventional pasture, soils were mapped as a Captina silt loam, Nixa, Savannah (fine-loamy, siliceous, semiactive, thermic Typic Fragiudults), and Johnsburg silt loam. Both sides have wetter regions, which are classified as fine, mixed, active, thermic Typic Endoaqualfs. According to the Koppen-Geiger classification^60^, the climate is humid subtropical (Cfa). Mean (30 year) annual precipitation is 1183 mm and mean annual temperature is 14.4 °C^61^.

### Field management and treatment implementation

In 1999, sixteen east–west oriented tree rows were established with a 15 m spacing. Initially, tree rows included northern red oak (*Quercus rubra* L.), eastern black walnut (*Juglans nigra* L.), and pecan [*Carya illinoinensis* (Wangenh). K. Koch]. In 2014, the rows formed by eastern black walnut trees were replaced by American sycamore (*Platanus occidentalis* L.), cottonwood (*Populus deltoides* W. Bartram ex Marshall), and pitch/loblolly hybrid pine (*Pinus rigida* × *P. taeda*). The alleys between rows were seeded with two forage species, including a cool-season species [orchardgrass, which was seeded at 17 kg pure live seed (PLS) ha^−1^ in Fall 2015, or a native warm-season mix [8:1:1 big bluestem (*Andropogon gerardii* Vitman), little bluestem (*Schizachyrium scoparium* {Michx. Nash} and indiangrass (*Sorghastrum nutans* L.)], seeded at 10 kg PLS ha^−1^ in Spring 2016. A Haybuster 107C no-till drill (DuraTech, Jamestown, ND) was used to plant the alleys. Cornerstone® Plus (N-[phosphonomethyl] glycine) was used prior to forage establishment at a 2.2 kg ha^−1^ rate (41% a.i.) to kill the existing vegetation. Thereafter, alleys were treated with Plateau (ammonium salt of imazapic) at 0.28 kg ha^−1^ rate (23.6% a.i.). Between 2001 and 2007, 3.9–6.7 Mg ha^−1^ of poultry litter (2–3% N) was broadcast-applied to the east half of the silvopasture each Spring. Further information on silvopasture site establishment and management practices can be found elsewhere^36^.

The conventional pasture system was established in 2019 to mirror the silvopasture system, and has been a grazing pasture for at least 20 years. The native warm-season grass mix and the orchardgrass were planted May 2019 and March 2020, respectively, using the same seeding rates and equipment as described for the silvopasture system. The entire paddock (3.3 ha) was fertilized with 67 kg N ha^−1^ as urea (CH_4_N_2_O) in March 2020 and March 2021. The orchardgrass was sprayed with Cimarron max (metsulfuron methyl) at 1.15 kg ha^−1^ (0.75%) rate on April 17, 2021, and the native mix was sprayed with Plateau at 0.28 kg ha^−1^ (23.6% a.i.) rate on May 5, 2021^35,62^. Between 2017 and 2019, 4.9 Mg ha^−1^ of poultry litter (84 kg N ha^−1^) was applied to selected alleys of both silvopasture and conventional pasture systems each Spring (Supplementary Fig. 1).

### Data collection

Soil cores (*n* = 15) were uniformly collected per system in March 2020, 2021, and 2022, covering the whole area of both systems (36.0904–36.0925°N; 94.1875–94.1914°W). Soil samples were collected in March (early Spring) to better reflect soil nutrient status prior to forage fertilization and regrowth. Samples were taken to depths of 0–15 cm using a 2 cm-diameter push probe, and subsequently ground and sieved to < 2 mm. Bulk density (BD) was determined by the core method^63^ on January 9, 2023, and considered to represent soil bulk density of 2022. Total C and N were determined via combustion using a VarioMax CN analyzer (Elementar Americas, Mt. Laurel, NJ). Since these soils do not contain carbonates based on previous studies in both sites, the total C analyzed corresponds to soil organic C (SOC). Soil C (Mg ha^−1^) and N (kg ha^−1^) stocks were calculated as described in Shi et al. (2018)^5^, using the 2022 BD values. Briefly, stocks (Mg ha^−1^) are obtained by multiplying the nutrient concentration (%) by the soil bulk density (Mg m^−3^) in the measured soil depth (0–15 cm). Soil pH and EC were measured on a 1:10 (soil:water) sample extraction^64^. Mehlich-3 extractable soil element concentrations were determined using a 1:10 soil mass:extractant solution volume ratio^65^ and analyzed by inductively coupled argon-plasma spectrometry (ICP, Agilent Technologies, Santa Clara, CA).

Soil temperature was monitored by type-T thermocouples installed 4.5 cm beneath the soil surface in the alleys approximately 4 m from the oak tree row. Soil volumetric water content was monitored by a CS-655 water content reflectometer (Campbell Scientific, Logan, UT) installed 3 cm beneath the soil surface offset from the thermocouple by 15 cm. These measurements were replicated in two locations in the silvopasture beneath the oak tree canopy and in two locations in the open pasture at approximately the same topographic location as the silvopasture. Measurements were taken every 30 s and average values were recorded every hour on a data logger (CR10X, Campbell Scientific). Microclimate measurements were recorded year-round, but only the data recorded between June 7 and July 11, 2022, were retrieved for this study.

Forage mass was determined per experimental unit (system and forage species) throughout the summer mob grazing period (June and July). Forage samples were collected during four dates in 2022 (May 31, June 9, June 16, and June 27). These dates allowed for investigating the contrasting growth patterns of cool- and warm-season grasses in response to variations in light, temperature, and soil moisture. During those sampling dates, three 0.25 m^2^ samples were collected from the grazed areas at 6 cm above-ground and geo-referenced. Quadrants were placed in the middle of the alleys (or in open pasture) per experimental unit to emulate available forage. Then, forage samples were weighed, dried at 70 °C for 48 h, and reweighed to determine moisture content. After drying, samples were ground using a Wiley mill (Tomas Scientific, Swedesboro, NJ) and sieved to < 1 mm. Total C and N in the forage samples were determined via high-temperature combustion, as described above. Lignin, acid-detergent fiber (ADF), and neutral detergent fiber (NDF) were determined using an ANKOM 2000 Fiber Analyzer (ANKOM Technologies, Macedon, NY45). Crude protein (CP) was calculated by multiplying percent N by 6.25. Total ash was determined based on ASTM standard E1755-0147. One gram of prepared forage tissue (sieved to < 1 mm) was placed in an oven-dried, porcelain crucible overnight at 105 °C. Crucibles were placed in a muffle furnace at 575 °C for 4 h. After 4.5 h, crucibles were removed and cooled to room temperature in a glass desiccator. The remainder material retained in the crucible was weighed, with ash concentration expressed as %^19,35^.

Twenty Angus crossbred heifers (*Bos taurus*) freely grazed the silvopasture and conventional pasture systems, ten per system, from June 7 to July 11, 2022 [2.56 and 2.51 animal units (AU) ha^−1^, respectively]. This short grazing period known as summer ‘mob grazing’ is typical for the US Mid-south, where pastures consist mostly of tall fescue, which is a cool season species and leaves a ‘summer slump’^20^. A beef mineral supplement was freely available during the grazing period, and no other feed supplement was provided to the animals. Nineteen GPS collars (Models Litetrack and 3300LR, Lotek Wireless Inc., Newmarket, ON) outfitted with one temperature sensor per collar were fitted on heifers June 6 to monitor cattle temperature every 15 s. Heifers were weighed prior to entering the silvopasture and conventional pasture systems on June 6, then on July 2 and July 12, 2022.

Land equivalent ratio (LER) is a mathematical tool used to elucidate the value of intercropping practices relative to monoculture production by indicating the amount of monoculture land required to achieve the yields of the intercropping system^24,26^. In this study, LER was used to calculate the productivity of the silvopasture relative to the conventional pasture system. LER is calculated by summing (1) the forage or livestock yield in the silvopasture divided by the forage or livestock yield in a comparable pasture and (2) the tree yields (e.g., timber or nuts) in the silvopasture divided by the tree yields of a comparable forest or orchard reference, as follows:

$${\text{LER}} = \left( {\frac{{\text{Forage yield or cattle weight silvopasture}}}{{\text{Forage yield or cattle weight pasture only}}}} \right) + \left( {\frac{{\text{Tree yield silvopasture}}}{{\text{Tree yield forest}}}} \right)$$

LER were calculated for each tree stand of the silvopasture system. Forage yields and cattle weight gains were collected in both systems in 2022, as described above. Tree diameter at breast height (DBH; 137 cm above soil level) was measured in 2021 for cottonwood, sycamore, pine, and northern red oak, and used as metric of tree yield in the silvopasture. Pecan nuts were harvested in 2022 and nut yield was estimated for 105 trees. The forest reference yields, that is, timber or nut yields of forest only systems, was obtained from the literature for each tree species^24,30–33^.

All methods were performed in accordance with the relevant guidelines and regulations. All methods were conducted in accordance with the recommended ARRIVE guidelines (Animal Research: Reporting of In Vivo Experiments; https://arriveguidelines.org/). All experimental protocols were approved by the Institutional Animal Care and Use Committee at the University of Arkansas. The use of plants in the present study complies with international, national, and institutional guidelines and follows the IUCN Policy on Research Involving Species at risk of Extinction and the Convention on the Trade in Endangered Species of Wild Fauna and Flora.

### Soil health assessment

The Soil Management Assessment Framework (SMAF)^27^ was used as an integrated approach to investigate the impacts of each system on soil quality (SQ) indicators and overall soil quality using soil samples collected in 2022. Seven soil indicators, namely BD, TOC, pH, EC, and extractable K and P, were included in the assessment. This approach is aligned with the recommendation of minimum of five indicators with at least one each representing soil biological, physical and chemical properties and processes^66^. Measured values of individual indicators were converted into scores between 0 and 1 using established algorithms in Excel, with 0 representing the lowest SQ value and 1 indicating the largest SQ value for each indicator. The algorithms, or scoring curves, are modified by SMAF factor classes (Supplementary Table 3), which account for inherent soil properties, climatic factors, site management, and selected analytical methods for soil chemical properties^67^. After conversion, the individual scores were integrated into an overall, percentage-based SQ index using simple addition^68^.

### Statistical analyses

Analysis of variance (ANOVA) of soil properties collected between 2020 and 2022 was performed using the SAS MIXED procedure^69^, with system (silvopasture vs. conventional pasture) as fixed effect and year in the repeated measures. The same procedure was used to compare cattle weight in each system across dates. ANOVA of forage quality components and yields considered system, forage species, and sampling dates as fixed effects, and replications as the random effect. When main effects or interactions were found between the explanatory factors, mean separation was performed by the SAS macro “pdmix800”^70^, with Fisher's least significant difference and Type I error rate of 5%. Student’s t-test was used to compare soil bulk density, SOC and N stocks, SMAF scores, soil temperature and volumetric water content, cattle temperature between systems and within time intervals (00:00–5:59, 6:00–11:59, 12:00–17:59, and 18:00–23:59; arbitrarily defined), and weight gains between systems. All statistical analyses were performed using SAS or the R (V. 4.2.2) software^71^.

### Supplementary Information

Supplementary Information.

## Data Availability

The datasets used and/or analyzed during the current study are available from the corresponding author on reasonable request.
