# Supplementary material for: Temperate silvopastures provide greater ecosystem services than conventional pasture systems
Source: Sci Rep. 2023 Oct 31;13:18658. doi: 10.1038/s41598-023-45960-0 (PMC10618464; doi:10.1038/s41598-023-45960-0)
Supplement: Supplementary file 1 — Supplementary Information. [file 41598_2023_45960_MOESM1_ESM.pdf]

SUPPLEMENTARY INFORMATION

Temperate Silvopastures Provide Greater Ecosystem Services than Conventional Pasture Systems

Helen C.S. Amorim[1,2]*, Amanda J. Ashworth[1], Peter L. O'Brien[3], Andrew L. Thomas[4], Benjamin R. K. Runkle[5], Dirk Philipp[6]

[1] *USDA ARS Poultry Production and Product Safety Research Unit, 1260 W. Maple Street, Fayetteville, AR, 72701, USA*

[2] *University of Arkansas, Department of Crop, Soil, and Environmental Sciences, 115 Plant Sciences Building, Fayetteville, AR, 72701, USA *Corresponding author (hamorim@uada.edu)*

[3] *USDA ARS National Laboratory for Agriculture and The Environment, 1015 N. University Blvd., Ames, IA, 50011, USA*

[4] *University of Missouri, Division of Plant Science and Technology, Southwest Research Extension and Education Center, Mt. Vernon, MO, 65712, USA*

[5] *University of Arkansas, Department of Biological and Agricultural Engineering, 231 ENGR Hall, Fayetteville, AR, 72701, USA*

[6] *Division of Agriculture, Department of Animal Science, AFLS B114, University of Arkansas, Fayetteville, AR 72701, USA*

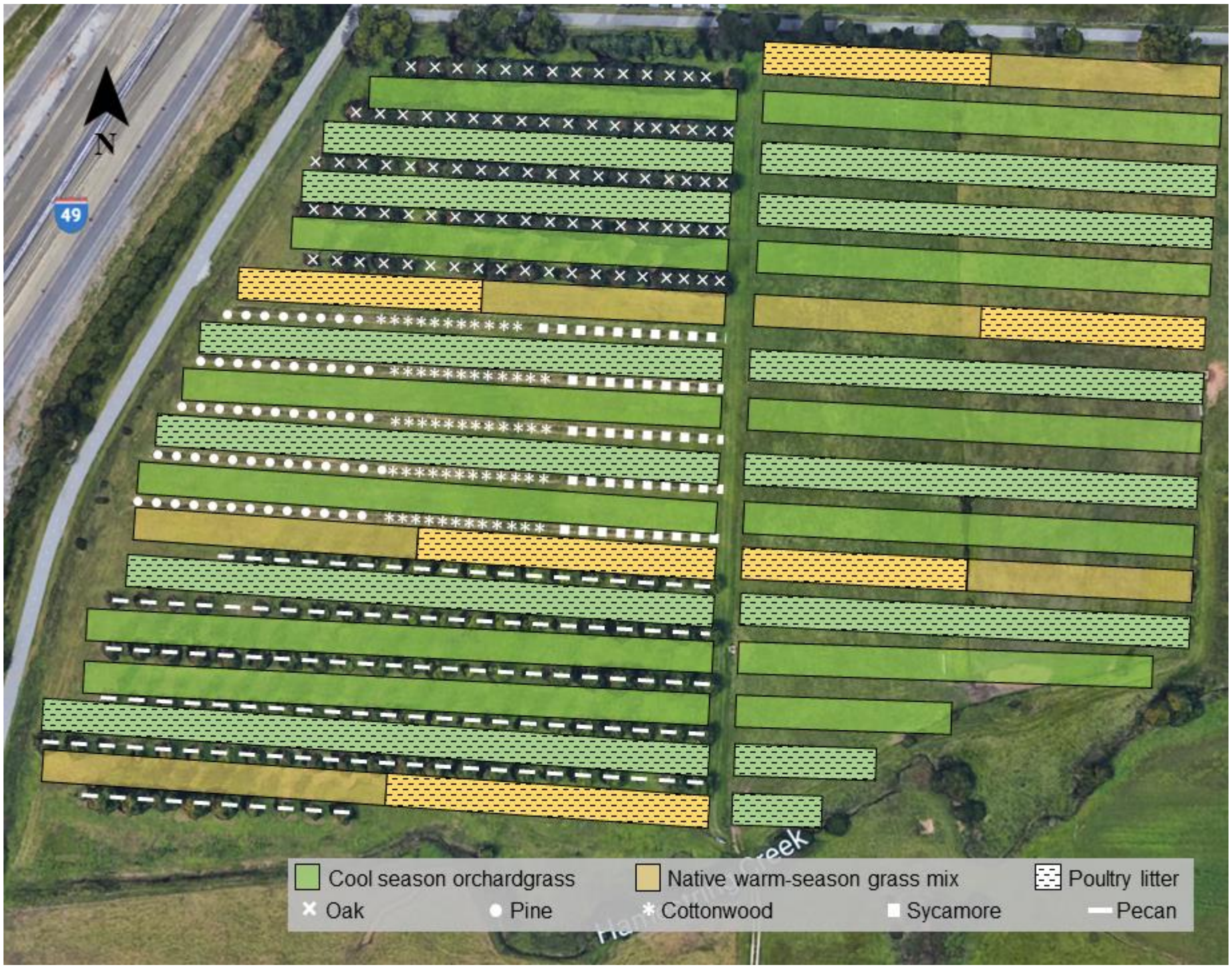

Supplementary Figure 1. Schematic representation of silvopasture and conventional pasture systems. Silvopasture (left) and conventional pasture (right) systems located at the University of Arkansas Agricultural Research and Extension Center in Fayetteville, AR. Symbols identify tree rows, and polygons identify grass species planted in tree alleys. Silvopasture and conventional pasture alleys include orchardgrass and a native warm-season grass mix. Dashed polygons identify alleys that received poultry litter (84 kg N ha^{-1}) between 2017 and 2019. Aerial image obtained from Google Earth.

Supplementary Table 1. Analysis of variance summary of effects of system (silvopasture and conventional pasture), time (2020, 2021, and 2022), and their interactions on selected Mehlich-3 soil properties at the 0-15 cm soil depth of the silvopasture and conventional pasture systems in Fayetteville, AR (*n* = 15).

**Source of variation**	**Soil properties†**									
	SOC	N	C:N	pH	EC	Ca	Mg	K	P	S
System	0.0277	0.4012	<0.0001	0.0130	0.0555	<0.0001	0.4536	0.0207	0.8932	0.6896
Time	0.8961	0.0390	<0.0001	0.0057	0.3052	0.0932	0.0124	0.3172	0.0404	<0.0001
System × Time	0.8012	0.9773	0.0290	0.2383	0.5071	0.3719	0.1651	0.6396	0.1244	0.9286

† SOC, soil organic C; C:N, carbon-to-nitrogen ratio; EC, electrical conductivity.

Supplementary Table 2. Mean values ± standard errors for C and N contents, C:N ratios, acid- (ADF) and neutral detergent fiber (NDF), lignin, crude protein (CP), and ash contents (%, except for C:N ratios) in forage samples [cool-season orchardgrass (OG) and native warm-season mix (NG)], collected in four sampling dates (May 31, June 9, June 16, and June 27, 2022) in silvopasture and conventional pasture systems in Fayetteville, AR (*n* = 3-11).

**System**	**Forage species** †	**Forage composition** ‡							
		C	N	C:N ratio	ADF	NDF	Lignin	CP	Ash
		May 31							
Silvopasture	OG	42.9 ± 0.2 a¶	1.6 ± 0.1 bc	27.5 ± 1.2 ef	36.8 ± 0.7 fg	63.8 ± 1.0 c-g	5.9 ± 0.8 f	10.1 ± 0.4 bc	1.5 ± 0.2 a
	NG	42.9 ± 0.2 a	1.9 ± 0.1 a	22.6 ± 1.2 g	38.4 ± 0.7 ef	64.2 ± 1.0 b-e	6.5 ± 0.8 ef	12.3 ± 0.4 a	1.9 ± 0.2 a
Pasture	OG	43.6 ± 0.2 a	1.6 ± 0.1 bc	28.4 ± 1.2 def	35.9 ± 0.7 g	62.1 ± 1.0 e-h	11.0 ± 0.8 abc	9.9 ± 0.4 bc	1.1 ± 0.2 a
	NG	43.9 ± 0.2 a	1.7 ± 0.1 b	26.3 ± 1.2 f	30.1 ± 0.7 h	63.1 ± 1.0 e-h	6.7 ± 0.8 ef	10.7 ± 0.4 b	1.1 ± 0.2 a
		June 9							
Silvopasture	OG	42.1 ± 0.2 a	1.6 ± 0.1 c	27.9 ± 1.2 ef	38.6 ± 0.7 ef	62.9 ± 1.0 e-h	9.7 ± 0.8 bcd	9.7 ± 0.4 c	1.4 ± 0.2 a
	NG	42.4 ± 0.2 a	1.3 ± 0.1 e-h	32.7 ± 1.2 abc	39.2 ± 0.7 de	61.3 ± 1.0 fgh	8.2 ± 0.8 de	8.4 ± 0.4 e-h	1.6 ± 0.2 a
Pasture	OG	43.3 ± 0.2 a	1.5 ± 0.1 c-f	30.1 ± 1.2 b-e	37.5 ± 0.7 efg	62.0 ± 1.0 e-h	10.2 ± 0.8 bcd	9.1 ± 0.4 c-f	2.8 ± 0.2 a
	NG	42.6 ± 0.2 a	1.5 ± 0.1 c-g	30.1 ± 1.2 b-e	38.8 ± 0.7 e	60.5 ± 1.0 h	12.9 ± 0.8 a	9.1 ± 0.4 c-g	2.4 ± 0.2 a
		June 16							
Silvopasture	OG	42.7 ± 0.2 a	1.5 ± 0.1 cde	29.5 ± 1.2 c-f	40.9 ± 0.7 cd	66.4 ± 1.0 abc	11.4 ± 0.8 ab	9.3 ± 0.4 cde	1.6 ± 0.2 a

	NG	43.1 ± 0.2 a	1.3 ± 0.1 fgh	34.2 ± 1.2 a	43.8 ± 0.7 a	68.1 ± 1.0 a	9.8 ± 0.8 bcd	8.1 ± 0.4 fgh	1.2 ± 0.2 a
Pasture	OG	43.0 ± 0.2 a	1.3 ± 0.1 fgh	33.2 ± 1.2 ab	41.3 ± 0.7 bc	63.8 ± 1.0 b-g	8.9 ± 0.8 cd	8.2 ± 0.4 fgh	1.1 ± 0.2 a
	NG	43.1 ± 0.2 a	1.4 ± 0.1 d-h	31.8 ± 1.2 a-d	40.7 ± 0.7 cd	64.1 ± 1.0 b-f	10.1 ± 0.8 bcd	8.6 ± 0.4 d-h	0.9 ± 0.2 a
		June 27							
Silvopasture	OG	42.4 ± 0.2 a	1.3 ± 0.1 fgh	33.4 ± 1.2 ab	42.8 ± 0.7 ab	66.7 ± 1.0 ab	11.9 ± 0.8 ab	8.1 ± 1.5 fgh	1.6 ± 0.2 a
	NG	42.1 ± 0.2 a	1.5 ± 0.1 cd	28.9 ± 1.2 def	41.1 ± 0.7 bcd	63.3 ± 1.0 d-h	11.6 ± 0.8 ab	9.5 ± 0.4 cd	0.9 ± 0.2 a
Pasture	OG	42.5 ± 0.2 a	1.3 ± 0.1 gh	34.4 ± 1.2 a	41.6 ± 0.7 bc	61.2 ± 1.0 gh	11.7 ± 0.8 ab	8.1 ± 0.4 gh	1.1 ± 0.2 a
	NG	42.5 ± 0.2 a	1.2 ± 0.1 h	35.2 ± 1.2 a	41.2 ± 0.7 bc	66.1 ± 1.0 a-d	10.7 ± 0.8 bc	7.6 ± 0.4 h	1.0 ± 0.2 a
p-value		*0.1310*	*0.0012*	*0.0021*	*<0.0001*	*0.0039*	*0.0003*	*0.0012*	*0.1242*

† OG, orchardgrass; NG, native warm-season mix;
‡ ADF and NDF, acid- and neutral detergent fiber; CP, crude protein;
¶ Means followed by the same letter within a column do not differ ($p < 0.05$).

Supplementary Table 3. SMAF algorithms and site-specific factors used to calculate individual and overall soil quality indices based on soil properties collected at the 0-15 cm soil depth in the silvopasture and conventional pasture systems in Fayetteville, AR, in 2022

Indicator†	Algorithm	Constant	Site-specific factors
SOC	$y = a/[1 + b \times \exp(-c \times SOC)]$	a = 1.0; b = 50.1	c = f (organic matter class "4" – low,<br>texture class "3" – silt loam, climate class "3" - ≤170 ˚C d and ≥ 550 mm
BD	$y = a - b \times \exp(-c \times BD^d)$	a = 0.994	b, c, d = f (texture class "3" – silt loam, mineralogy class "3" – other than smectite and glassy)
pH	$y = a \times \exp[-(pH - b)^2/(2c^2)]$	a = 1.0	b, c = f (crop code "3" – tall fescue)
EC	If $EC_{sat} \leq 0.3$, then $y = 3.33 \times EC_{sat}$;<br>if $0.3 < EC_{sat} < T$, then y = 1;<br>if $EC_{sat} > T$, then $y = m \times EC + b$		T^b, b, m = f (crop code "3", texture class "3")
P	If P ≤ max (for crop and method), then $y = (ab+c \times P^d)/(b + P^d)$; if P > max (for slope and method), then $y = a - b \times \exp(-c \times P^d)$, and y = 1	$a = 9.26 \times 10^6$; c = 1.0; d = 3.06	b = f (crop code "3", TOC content, texture class "3", method "2" – Mehlich 3, slope class "2" – 2-5%, weathering class "3" – slightly weathered)
K	$y = a[1 - \exp(-b \times K)]$	a = 1.05;<br>b = -0.0098	a, b = f (crop code "3", texture class "3")

† SOC, soil organic C; BD, bulk density; EC, electrical conductivity; Tb, crop-specific threshold beyond which yield decreases are expected to occur.
